# Occurrence, antimicrobial resistance and genomic features of *Klebsiella pneumoniae* from broiler chicken in Faisalabad, Pakistan

**DOI:** 10.3389/fvets.2024.1433124

**Published:** 2024-08-19

**Authors:** Muhammad Moman Khan, Muhammad Ahmed Mushtaq, Nayyar Abbas, Fariha Fatima, Marjorie J. Gibbon, Peter Schierack, Mashkoor Mohsin

**Affiliations:** ^1^Institute of Biotechnology, Brandenburg University of Technology Cottbus-Senftenberg, Senftenberg, Germany; ^2^Institute of Microbiology, University of Agriculture, Faisalabad, Pakistan; ^3^Department of Life Sciences, The Milner Centre for Evolution, University of Bath, Bath, United Kingdom

**Keywords:** antimicrobial resistance, poultry, genomics, *K. pneumoniae*, sequence type

## Abstract

**Introduction:**

The dissemination of antimicrobial resistance (AMR) in critical priority pathogens is a significant threat. Non-clinical reservoirs of AMR, such as agriculture and food production facilities, may contribute to the transmission of clinically relevant pathogens such as multidrug-resistant (MDR) *Klebsiella pneumoniae*. There is currently very limited knowledge regarding the population structure and genomic diversity of *K. pneumoniae* in poultry production in Pakistan.

**Methods:**

We explored healthy broilers in a commercial farm from Faisalabad, Pakistan, and identified six *K. pneumoniae* strains from 100 broiler birds. We characterized the strains, determining clonality, virulence and antimicrobial resistance genes using next generation sequencing.

**Results:**

The evaluation of antimicrobial susceptibility revealed that all the strains were MDR. Genomic analysis showed that 3/6 strains belonged to ST152, harbouring acquired resistance aminoglycosides [*aadA2*, *aph(4′)-Ia*], β-lactams (*bla_SHV-187_*, *bla_LAP2_*), fosfomycin (*fosA6*), tetracycline (*tetA*), trimethoprim (*dfrA12*), quinolone (*qnrS1*), sulphonamides (*sul2*) and phenicol (*floR*). All the strains harboured the efflux pump genes *oqxA*, *oqxB*, *emrR*, *kpnG*, *kpnH*, *kpnF*, *baeR*, *mtdB* and *mtdC*. All six strains encoded identical virulence profiles possessing six genes, i.e., *ureA*, *iutA*, *entB*, *allS*, *fimH* and *mrkD*. Phylogenomic analysis of the dominant sequence type (ST152) present in our dataset with publicly available genomes showed that the isolates clustered to strains mainly from human sources and could pose a potential threat to food safety and public health.

**Discussion:**

The combination of these findings with antimicrobial use data would allow a better understanding of the selective pressures that may be driving the spread of AMR. This is the first report of MDR *K. pneumoniae* isolated from broiler hens in Pakistan, and the finding suggests that routine surveillance of WHO critical priority pathogens in such settings would be beneficial to the development of effective control strategies to reduce AMR.

## 1 Introduction

*Klebsiella pneumoniae*, a member of the *Enterobacteriaceae*, is commonly carried in healthy humans and other animals (1) but is also an important cause of community- and hospital-acquired infections (2). The rise of antimicrobial resistance (AMR) in *K. pneumoniae* led to its inclusion as an ESKAPE pathogen, organisms that are increasing threats to public health (3), as well as being on the World Health Organisation (WHO) bacterial priority pathogens list (4). In addition, a hypervirulent phenotype has emerged, in particular in East and Southeast Asia, with increasing reports of convergent strains carrying both AMR and hypervirulence (5). With an open pangenome and large accessory genome, including many genes likely to have been acquired from other bacterial taxa, *K. pneumoniae* has great genomic plasticity and acquisition of resistance and virulence determinants is common (6); *K. pneumoniae* is recognised as an important source of AMR globally (7).

The ‘One Health’ approach, which recognises the interconnection between human, animal and environmental health, lays bare the links between use of antibiotics and the spread of AMR within and between the three domains (8). The presence of *K. pneumoniae* in healthy animals, particularly ones in the human food chain, is one possible source for the spread to humans. *K. pneumoniae* has been shown to be common in the intestinal flora of poultry grown for human consumption in Norway (9) and Portugal (10). In these countries, where the use of antibiotics is low, isolates of *K. pneumoniae* were found that were either hypervirulent or multidrug-resistant (MDR), including some in Portugal that were identical to clinical isolates. In contrast, in Pakistan there have been high levels of antibiotic use in poultry production, for treatment, prophylactic, and growth promotion purposes (11, 12). Such overuse of antibiotics can lead to an increase in AMR, with a clear risk to human health (13).

MDR *K. pneumoniae* and isolates carrying virulence genes have been documented in human infections in Pakistan (14). However, no studies have investigated the presence of AMR in *K. pneumoniae* in poultry produced for human consumption, which is likely to be an important reservoir for the spread to humans (15). Since the inception of the Pakistan National Action Plan on AMR (11), little effort has been made to investigate this WHO critical priority pathogen in food animals such as the broiler chicken. In this study we report the presence of *K. pneumoniae* in broiler chickens on a single farm in Faisalabad, Pakistan. Using phenotypic antimicrobial susceptibility and whole genome sequencing to characterise the isolates, we focus on clinically relevant AMR genes, MLST and serotypes.

## 2 Materials and methods

### 2.1 Isolation and identification of *K. pneumoniae*

This pilot observational study was conducted to detect MDR *K. pneumoniae* and investigate its phenotypic and genomic features in a commercial poultry farm in Faisalabad, Pakistan. During winter 2023, a total of 100 cloacal swabs from broilers birds were screened. In order to selectively isolate and screen *K. pneumoniae*, cloacal swabs were spread onto Simmons citrate agar (SCAI, Oxoid) with amoxicillin (10 μg/mL) and myo-inositol (10%) and incubated at 37°C for 48 h. Possible *K. pneumoniae* isolates were streaked on CHROMagar orientation plates (Mast Diagnostica GmbH, Reinfeld, Germany) and blue coloured pure colonies were subjected to MALDI-TOF (microflex, Bruker, Billerica, MA, United States) for confirmation.

### 2.2 *In vitro* antimicrobial susceptibility testing

The antimicrobial susceptibility was determined using the VITEK^®^2 at the Laboratory Diagnostics and Microbiology of Klinikum Niederlausitz GmbH. In case of tigecycline, minimum inhibitory concentration (MIC) of six strains was determined on a range of concentrations (0–32 μg/mL) using broth microdilution method. Three technical and biological repetitions were performed for each strain and *E. coli* strain ATCC 25922 was used as control. MDR was defined as resistance to three or more antibiotics of different classes (16). Results were interpreted according to the breakpoints of Clinical and Laboratory Standards Institute documents and European Commission on Antimicrobial Susceptibility Testing (EUCAST) documents (17, 18).

### 2.3 Whole genome sequencing and analysis

Genomic DNA was extracted from an overnight culture (2 mL) of *K. pneumoniae* strains with QIAcube automated system (Qiagen). DNA concentration was measured using Qubit (ThermoFisher Scientific, United States) and sent for commercial sequencing to Azenta Life Sciences, Leipzig, Germany. The Illumina NovaSeq platform carried out paired-end sequencing, generating 150 bp pair end reads and resulting in a genome coverage of 100x. The initial processing of the raw reads involved quality trimming and removal of Illumina-specific adapters with the Trimmomatic (19). For quality assessment, the sequences were further analysed using FastQC, and all samples exhibited a quality score not less than 30. The genome was assembled using SPAdes (20) and further assessed for assembly quality via Quast (21). The contigs were annotated using Prokka (22) for the identification of virulence genes and factors. Antibiotic resistance genes (ARGs) were ascertained by utilizing the Comprehensive Antibiotic Resistance Database (CARD) (23) and ABRicate^1^ (24). Plasmids were identified from each of the WGS using Staramr which employs PlasmidFinder database (25, 26). Furthermore, Kleborate v2.2 (5) was used identify *Klebsiealla*–specific information on pathogenicity and virulence genes and to determine ST. Virulence genes (*n* = 19) involved in formucoviscosity, LPS synthesis, adhesins, allantoin metabolism, iron acquisition and siderophores, cytotoxicity and urease (27) were analysed using BLAST (28). The phylogenetic analysis was conducted by comparing dominant STs to global types found in published literature using RAxML version 8.2.12 by 100 bootstrap (29). Moreover, pangenome analysis through Roary (30) to identify core genome (4,278 genes) was performed and a maximum-likelihood phylogenetic tree with 100 bootstrap was constructed and visualized using iTOL (31).

## 3 Results

### 3.1 Occurrence and antibiotic susceptibility profiles of *K. pneumoniae*

**Figure 1:**
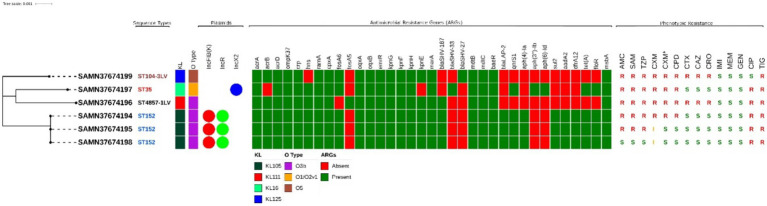
Maximum likelihood tree representing the phylogenetic relationship between 6 *K. pneumoniae* strains from this study. Associated metadata shown here represents the STs, KL and O-type identified using Kleborate, ARGs are determined through CARD database using Abricate. The phenotypic antimicrobial profiling for all antimicrobials except tigecycline is done by VITEK^®^2 illustrated as (R = Resistant, I = Intermediate, S = Sensitive). AMC, Amoxicillin + Clavulanic acid; SAM, Ampicillin + sulbactam; TZP, Piperacillin + Tazobactam; CXM, Cefuroxime; CXM*, Cefuroxime + Axetil; CPD, Cefpodoxime; CTX, Cefotaxime; CAZ, Ceftazidime; CRO, Ceftriaxone; IMI, Imipenem; MEM, Meropenem; GEN, Gentamicin; CIP, Ciprofloxacin; TIG, Tigecycline; FOS Fosfomycin; SXT, Trimethoprim + Sulfamethoxazol. All the metadata is illustrated using iTOL.

A total of 6 *K. pneumoniae* isolates were recovered from 100 chicken cloacal swabs. MICs of 16 antimicrobial agents showed that the strains had different resistance profiles, and all were designated as MDR (Figure 1). Most of the strains were resistant to second generation cephalosporins (cefuroxime and cefuroxime+axetil) and five of them were also resistant to penicillin and β-lactam in combination. Two of the strains (SAMN37674196 and SAMN3764199) showed resistance to third generation cephalosporins (cefotaxime, ceftazidime and ceftriaxone). Notably, tigecycline resistance was observed in all six strains. All the strains were susceptible to carbapenems (imipenem and meropenem) and aminoglycoside (gentamicin).

#### 3.1.1 Genetic divergence of *K. pneumoniae* strains

The genetic diversity of MDR-*K. pneumoniae* strains in our study were examined using MLST (32). Four STs were identified (ST104-3LV, ST35, ST4857-1LV and ST152), with ST152 being the predominant type, detected in three strains which also possessed the same plasmid types, i.e., IncFIB(K) and IncR, as shown in Figure 1.

### 3.2 Drug-resistance genes and virulence genes

Analysis of ARGs showed that the *K. pneumoniae* strains possessed a variety of genes conferring resistance to multiple antimicrobials (Figure 1). The number of ARGs detected in the strains ranged from 21 to 30. We did not detect any isolate harbouring resistance to carbapenems or colistin. Strains belonging to ST152 were found to encode resistance to aminoglycosides [*aadA2*, *aph(4′)-Ia*], β-lactams (*bla_SHV-187_*, *bla_LAP2_*), fosfomycin (*fosA6*), tetracycline (*tetA*), trimethoprim (*dfrA12*), quinolone (*qnrS1*), sulphonamides (*sul2*) and phenicols (*floR*). All the strains encoded the efflux pump genes, i.e., *oqxA*, *oqxB*, *emrR*, *kpnG*, *kpnH*, *kpnF*, *baeR*, *mtdB* and *mtdC*.

**Figure 2:**
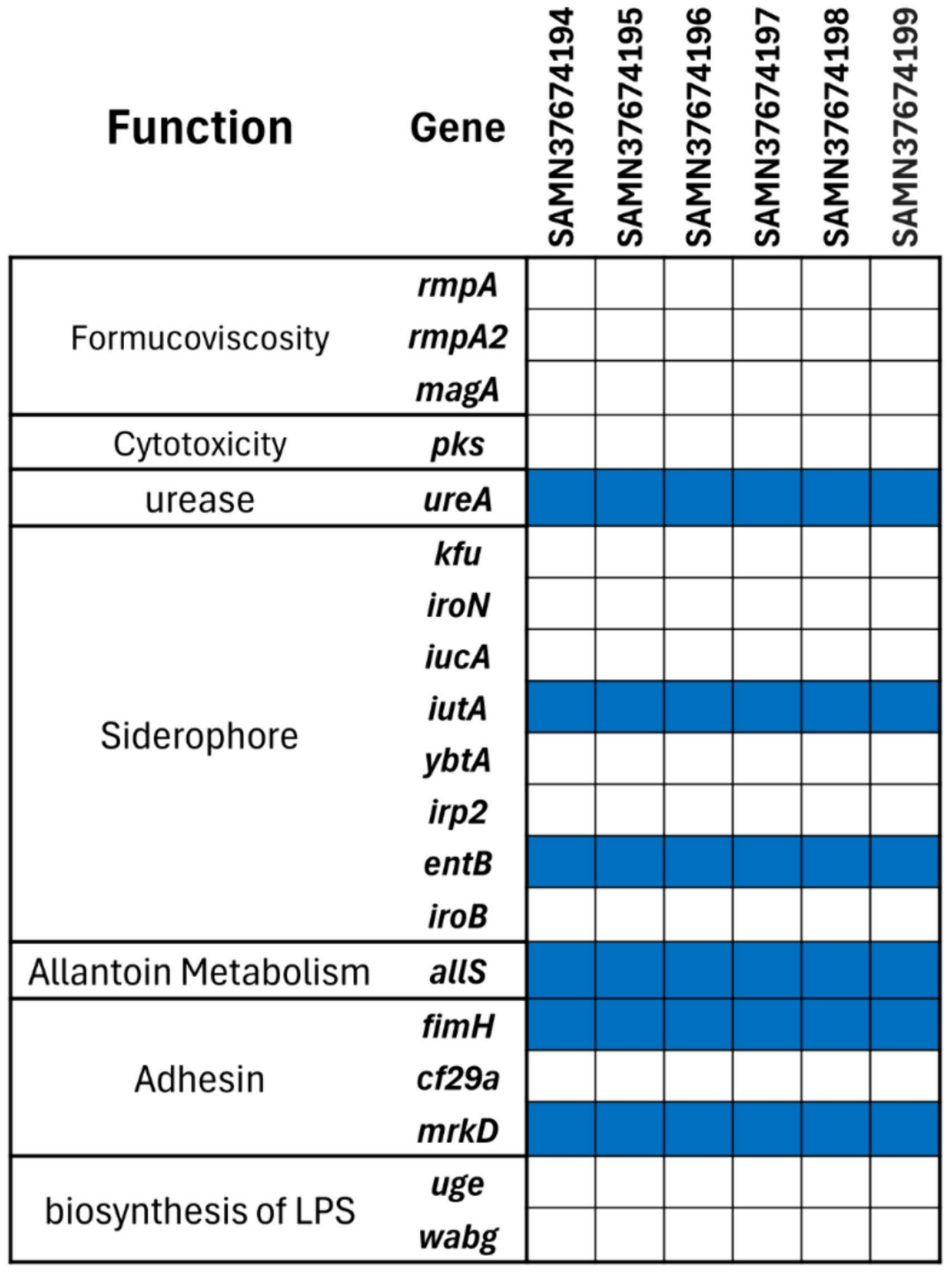
Virulence gene profiles of the six MDR *K. pneumoniae* strains. The blue tile in the figure shows presence and white tile exhibits the absence of the gene.

The strains were also analysed for major virulence encoding factors, resulting in identical profile as shown in Figure 2. A total of 19 factors were examined, only six genes were found. Notably, none of the strains possessed formucoviscosity genes (i.e., *rmpA*, *rmpA2*, *magA*) and LPS biosynthesis genes (i.e., *uge* and *wabG*). However, all strains encoded allantoin metabolism gene *allS* and urease encoding *ureA* gene. Only two of the genes encoding siderophores, which are also important virulence factors, were detected (i.e., *iutA* and *entB*). Similarly, only two out of three important adhesins (*fimH* and *mrkD*) were found among all six *K. pneumoniae* strains.

### 3.3 Global genomic epidemiology of ST152

**Figure 3:**
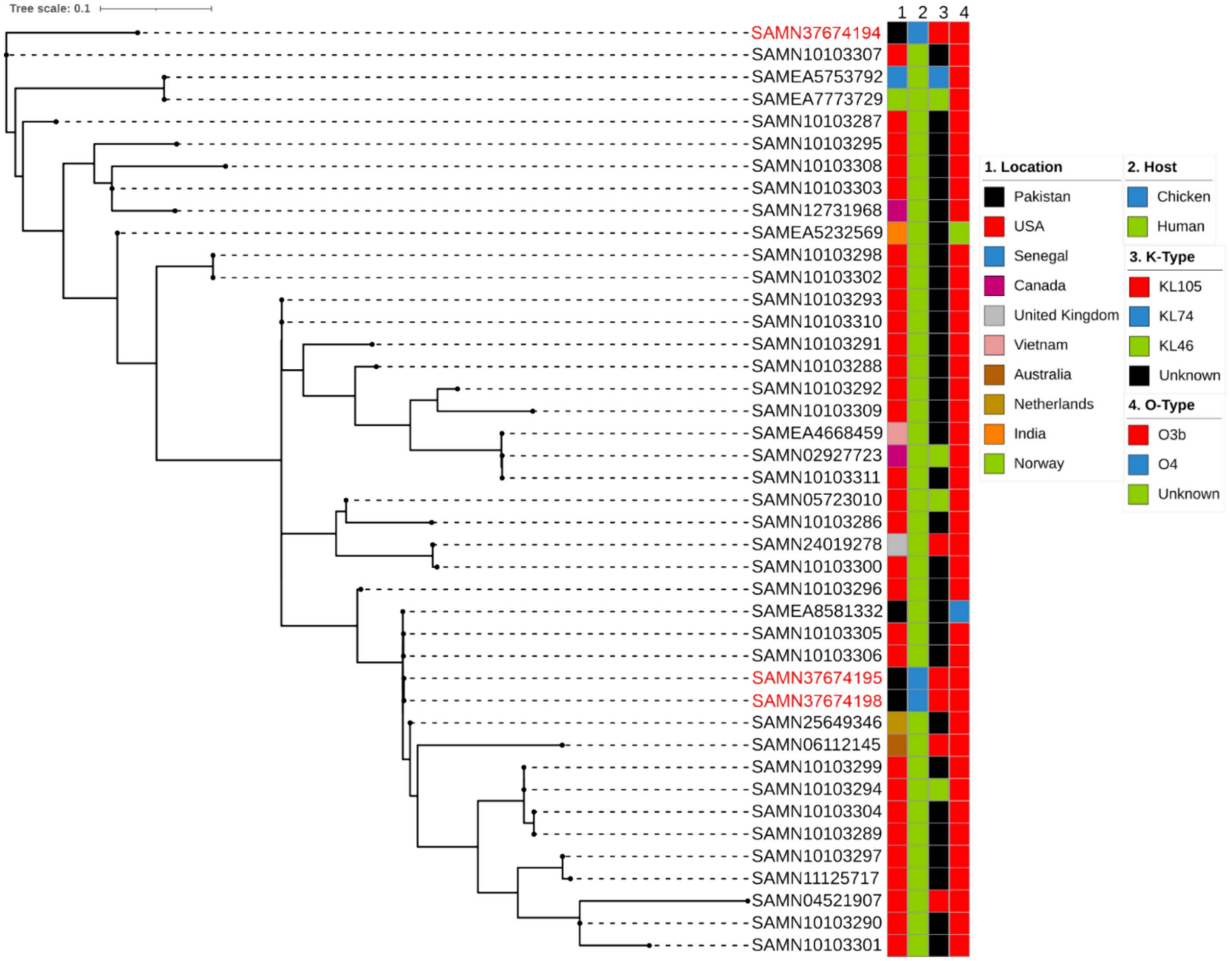
Phylogenetic comparison of the *K. pneumoniae* ST152 depicting location, host, K and O type.

In order to analyse global genomic epidemiology of ST152, we conducted a single nucleotide polymorphism (SNP)-based phylogenetic analysis to compare the genetic similarities and evolutionary relationships among the three ST152 *K. pneumoniae* isolates in our study and 39 other publicly available ST152 isolates. According to Pathogenwatch database, 196 strains have been reported belonging to ST152 with only one strain isolated from bovine and the rest were isolated from humans (33). We selected the 39 ST152 isolates used based on their location (Pakistan), K type or O type from the Pathogenwatch database. All 39 strains (Supplementary Table S1) were derived from humans, with 29 of them originating in United States (Figure 3). As a result, the strains investigated in our study were primarily classified into four clusters. This is the first report of ST152 being isolated from poultry, specifically chicken. All the isolates included in this analysis had O-type of O3b except one other human isolate reported from Pakistan with O-type of O4. In case of K-types of ST152, most of them were unknown but there were six strains of KL105 (three strains from the present study), four strains of KL46 and only one strain from Senegal typed as KL74. Isolates from this study were all categorized as KL105 (Figure 3).

## 4 Discussion

This study explores the occurrence, antimicrobial resistance, and genetic diversity of *K. pneumoniae* in healthy broiler chickens in Faisalabad, Pakistan. We isolated six *K. pneumoniae* strains from a total of 100 healthy broiler chicken. Two studies at poultry farms in China and Bangladesh reported recovery rates of *K. pneumoniae* of 4.67 and 43.9%, respectively, (34, 35), but both included environmental samples from the poultry farms and so are not directly comparable. A study of veterinary and agricultural waste in Pakistan found two isolates of MDR *K. pneumoniae* in sludge and wastewater from poultry farms (36).

All six of the *K. pneumoniae* isolates in this study are MDR. We observed high rates of resistance to multiple classes of antimicrobials including second generation cephalosporin (C CXM and CXM*) (83.3%), third generation cephalosporin (CTX, CAZ AND CRO) (33.3%) penicillin and β-lactam combination (66.6%), fluoroquinolone (83.3%), sulphonamide (50%) and tetracycline (100%). Resistance to all of these antimicrobials is of concern for human health (7). Resistance to third generation cephalosporins is a global problem; in Europe third-generation cephalosporin-resistant *K. pneumoniae* has been reported as the third largest cause of infections and attributable deaths (37). Assessing the health burden of infections with antibiotic-resistant bacteria in the EU/EEA (37). Piperacillin/tazobactam (TZP) is prescribed to immune-compromised and critically ill patients (38). Fluoroquinolones are used in clinical settings due to their broad-spectrum of activity, and resistance is increasing rapidly in *K. pneumoniae* (39). Tigecycline resistant strains of *K. pneumoniae* were first isolated in hospitals and have been increasingly reported since its introduction (7). Thus all of these resistance phenotypes are potentially a threat to human health.

We employed WGS to identify potential resistance mechanisms of MDR the *K. pneumoniae* isolates. The six isolates in this study encoded multiple MDR efflux pumps, which can reduce susceptibility in clinically significant pathogenic bacteria (40). One of the most important multidrug efflux systems, detected in five out six *K. pneumoniae* isolates, was acriflavine resistance B (AcrB) encoded by *acrAB*. This pump, belonging to resistance nodulation cell diving (RND) superfamily of efflux transporters, allows bacteria to exhibit resistance to various antimicrobial classes of antibiotics, i.e., quinolones, beta-lactams, tetracyclines, macrolides, aminoglycosides, and chloramphenicol (40, 41). *kpnEFGH* operon, also detected across six strains, with the exception of *kpnE* in SAMN37674197, encodes for a small multidrug resistant (SMR) type efflux pump (42). The KpnEF efflux pump confers resistance to a variety of antibiotics; antimicrobial susceptibility testing of the *kpnEF* deletion mutant of different *K. pneumoniae* serotypes established its role in broad-spectrum AMR (42). Another antibiotic efflux pump encoded by all the six strains was OqxAB. The overexpression of *oqxAB* confers resistance against not only multiple drugs such as quinoxalines, quinolones, tigecycline, nitrofurantoin and chloramphenicol but also detergents and disinfectants (43). *mdtBC*, encoding a RND-type drug exporter, stimulated by response regulator BaeR in *Escherichia coli* (44), were detected in all six *K. pneumoniae* isolates. MdtBC contains two different transporter proteins, MdtB and MdtC, and appears to function only as a B_2_C heterotrimer. When overexpressed, it pumps out norfloxacin, novobiocin, cloxacillin, and deoxycholate (45).

The isolates in this study were not hypervirulent, carrying only a small number of virulence determinants. Although all of the isolates had one gene from the locus encoding the siderophore aerobactin, the other genes were absent. The K and O loci in these isolates are not associated with clones known to pose a threat to human health (46).

Against a background of other ST152 isolates from Pakistan and with the same K- and O- loci, the two isolates SAMN37674195 and SAMN37674198 appear to be very similar to each other, and to other isolates from Pakistan and United States. SAMN37674194 is more distinct, being less closely related to the other ST152 isolates selected. It would be interesting to compare this isolate with a wider selection of *K. pneumoniae* isolates, perhaps including other, related, STs.

The other three STs represented in this study were ST104-3LV, ST35 and ST4857-1LV. For ST35, there have been reports of MDR isolates which are also hypervirulent, have acquired yersiniabactin (47, 48). None of the isolates in this study carry yersiniabactin, but has been identified in agricultural sources in Pakistan in recent years. In a study of dairy cows in three states in Pakistan, our group isolated *K pneumoniae* with otherwise low virulence profiles but from two sites some isolates did carry yersiniabactin (49).

Overall, we detected a variety of MDR *K. pneumoniae* strains harbouring important AMR genes in broiler hens in a single commercial farm in Faisalabad, Pakistan. Some of these strains were very similar to strains that have been found to cause disease in humans, highlighting the potential risk of spread of such strains from commercially produced poultry to humans. A useful addition to this investigation would be antimicrobial usage data from the farm, which could allow a better understanding of the drivers of the spread of the resistance genes. In addition, the use of short read sequencing did not allow a complete study of the plasmids found to be present in the strains, particularly the ST152 isolates. As AMR genes and virulence factors are often harboured on plasmids (6) better characterisation of them would be beneficial to our understanding of the risk of their spread. Despite these limitations, this study has provided a useful addition to our knowledge of this important pathogen in the poultry industry in Pakistan, further highlighting the need for a one-health approach to control of the spread of AMR.

## Data Availability

The data presented in the study are deposited in the online repository NCBI under the accession number PRJNA1023867. The datasets presented in this study can be found in online repositories. The names of the repository/repositories and accession number(s) can be found in the article/Supplementary material.

## Footnotes

^1^ https://github.com/tseemann/abricate
